# Importance of Conjugation of the Bile Salt on the Mechanism of Lipolysis

**DOI:** 10.3390/molecules26195764

**Published:** 2021-09-23

**Authors:** Natalia Łozińska, Christian Jungnickel

**Affiliations:** Department of Colloid and Lipid Science, Faculty of Chemistry, Gdańsk University of Technology, ul. Narutowicza 11/12, 80-233 Gdańsk, Poland; natlozin@student.pg.edu.pl

**Keywords:** bile salts, lipolysis, CMC, aggregation number, MSR

## Abstract

We aim to advance the discussion on the significance of the conjugation of bile salts (BS) in our organism. We hypothesize that conjugation influences the rate of lipolysis. Since the rate of lipolysis is a compound parameter, we compare the effect of conjugation on four surface parameters, which contribute to the rate. Since deconjugation is due to gut microbiota, we hypothesize that microbiota may affect the rate of lipolysis. A meta-analysis of literature data of critical micelle concentration, β, aggregation number, and molar solubilization ratio has been performed for the first time. In addition, critical micelle concentration (CMC), interfacial tension, and lipolysis rate measurements were performed. It was found that the unconjugated BS in mixed micelles increases the antagonism between the BS, therefore, increasing the CMC. This correlated with the effect of unconjugated BS on the solubilization capacity of mixed micelles. The collected literature information indicates that the role of the BS and its conjugation in our organism is a key factor influencing the functioning of our organism, where too high levels of unconjugated BS may lead to malabsorption of fat-soluble nutrients. The experimental lipolysis results irrevocably showed that conjugation is a significant factor influencing the rate.

## 1. Introduction

Bile salts (BS) are planar surfactants, which have methyl groups on the convex side and hydroxyl groups on the concave α-side [1]. These rigid amphiphiles [2] lack the typical flexibility of linear surfactants, which results in occasional flipping of the molecules so that the hydrophilic parts may remain inside the core, while hydrophobic parts may remain in water [3]. Self-assembly of BS is driven both by induced dipole interaction and hydrogen bonding between the BS molecules.

BS plays a crucial role in the digestion and absorption of nutrients, extraction of waste products from our body and are known to function as steroid hormones regulating nutrient metabolism [4]. The compounds originate as bile acids (BA) that are synthesized from cholesterol in the liver and stored in our gallbladder [5]. BA are conjugated in hepatocytes with either a molecule of glycine or taurine at the C-24 carboxyl group by amino acid N acyltransferase [4,6]. Conjugation is possible due to the conversion of the bile acids to their coenzyme (CoA) thioester [7]. Conjugation reduces the pK_a_ of the formed BS and increases their hydrophilicity. The pK_a_ value was measured to be 6 for the unconjugated BA, 4.5 for glycine conjugated BA, and 1.5 for taurine conjugated BA [8]. The conjugated form of the BA present in our gastrointestinal tract is therefore called bile salts (BS), as they are commonly deprotonated [9]. 

BS plays many important roles in our organism. One of these is their ability to create mixed micelles, which act as vehicles for a variety of molecules, including cholesterol in the liver, thereby becoming the major path of removal of cholesterol from our body [10]. In the small intestine, bile salt is responsible for decreasing the surface interfacial tension of lipid droplets, which promotes the emulsification of oil droplets [11]. Moreover, BS contributes to the adsorption/desorption process of lipase [12]. BS are responsible for the removal of the lipolysis products from the interphase, solubilizing them in the mixed micelles, which allows for transport from intestinal lumen across into the intestinal mucosa to the gastrointestinal epithelium (enterocytes) [13,14,15]. They are removed from the bloodstream by the active transporters on the sinusoidal membrane of hepatocytes and are secreted back into the bile [16]. After entering the bloodstream, BS are transported to the liver.

In addition, BS regulates the composition of gut microbiota [17] with their known antimicrobial activity. Part of the BS undergo deconjugation and create secondary BA: deoxycholic acid and lithocholic acid, due to bacterial action [4]. They also act as a signaling molecule by modulating the BS receptors FXR and TGR5 [16]. In the ileum, BS are absorbed and transported back by the portal vein to the liver. In the small intestine, the flow and reabsorption of the BS and secondary BA through our body is known as the enterohepatic recirculation process [18]. Emulsification, formation of mixed micelles, regulating gut microbiota, and binding to the Farnesoid X receptor (FXR) are influenced by the level of conjugated BS [19]. FXR signaling reduces the expression of cholesterol 7α-hydrolase (CYP7a1), a rate-limiting enzyme in bile acid synthesis, and as a consequence, primary BA synthesis is reduced when its level is already high [20]. Unconjugated BS will have a higher affinity to the FXR than conjugated [21], and this will consequently inhibit new BS synthesis [22].

However, the concentration and type of the BS present in our gastrointestinal tract depend on many different factors, acting simultaneously. The most significant factor controlling the level of conjugation of BS is the intestinal flora [23]. These bacteria, mostly Gram-positive (such as *Lactobacillus*, *Enteroccocus*, *Bifidobacterium*, *Clostridium*), as well as some Gram-negative (*Bacteroides* spp.) possess bile salt hydrolase (BSH) [20], which catalyze the deconjugation of the BS. There are five known transformation mechanisms of conjugated BS by intestinal bacteria: dehydroxylation, dehydration, and epimerization, and most reported recently, the amide conjugation of the cholate backbone with phenylalanine, tyrosine, and leucine [24] and deconjugation of the amino acids glycine or taurine [24,25]. Deconjugation is investigated here since it is the most well-studied transformation and is a prerequisite for further transformation with CYP7a1 [26,27].

It has been suggested that the disruption of the composition of the intestinal microflora will result in a change in the BSH activity [28]. This, in turn, leads to a number of diseases. Weight gain may result from the change in lipolysis, which is enhanced by the dysregulation of BA homeostasis, and consequent reabsorption of BS [29]. Higher levels of BS in the colon will lead to the development of colon cancer [30,31,32]. Cholelithiasis may form with higher levels of unconjugated bile by reducing the removal of cholesterol from the liver [33], resulting in gallstones, among others [16]. 

The aim of this paper, therefore, is to analyze the effect that changing conjugation has on the lipolysis rate, which is commonly expressed graphically. Therefore, we investigate four interface parameters, which directly influence the lipolysis rate. These parameters are CMC, MSR, BS interaction, and aggregate number. The analysis will lead to a better understanding of why changes in the level of conjugation of bile salts have such profound effects on our bodies. In addition, we will show experimentally how the level of conjugation influences the rate of lipolysis in in-vitro digestion experiments.

## 2. Results

Conjugation changes of bile salts in the small intestine, as shown in Table 1. As can be seen, the range of the average ratio of conjugated to unconjugated is 94:5% in the duodenum, 93:7% in the jejunum, and 82:18% in the ileum. This change in the ratio is due to the higher presence of BSH, which is found in Gram-positive bacteria that commonly reside in the ileum [34].

### 2.1. Micellization

Micellization of BS is a representative and often measured parameter, which provides information on which concentration of BS micelle will form, where a lower CMC would indicate a lower concentration of BS is required to form micelles.

Not much CMC data exists in literature dating back to 1962. Performing a meta-analysis on CMC data is hindered by the large variety of methods that are used to determine the CMC (such as potentiometric, calorimetric, or conductometric) and measurements being performed in various conditions. For this analysis, we assumed that each method was equally valid, and we looked only at sodium cholate and sodium chenodeoxycholate and their transformation products, measured at 298.25 K. Even though the temperature dependence of the CMC is weak [3], including another variable (such as temperature) into a meta-analysis will reduce the strength of conclusions that can be drawn.

**Figure 1:**
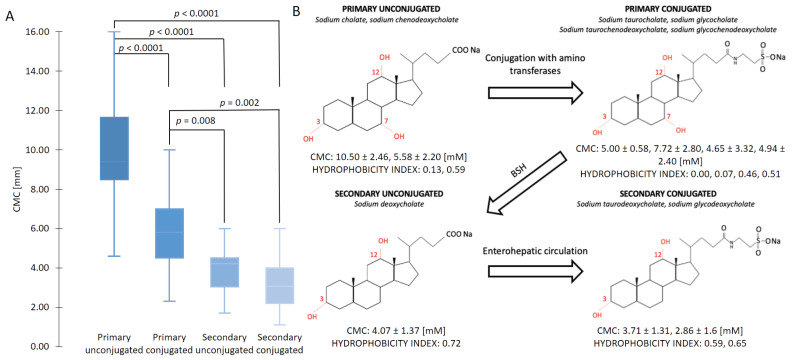
(**A**) Comparison of CMCs of conjugated/unconjugated, primary/secondary BS at the temperature of 298.15 K. The conjugated forms of the BS generally display a lower CMC than their respective unconjugated forms. (**B**) Structural changes are shown together with the average CMCs values of conjugated and unconjugated BS at 298.15 K as well as their hydrophobicity index. The hydrophobicity index values are taken from Heuman et al. [45]. The conjugated form of the BS is characterized by a smaller average CMC than their unconjugated form with a lower hydrophobicity index. The *p*-value of primary conjugated and secondary unconjugated was calculated to be 0.008.

Figure 1A shows the results of the CMCs determined by other groups. It can be seen that primary unconjugated sodium cholate and sodium chenodeoxycholate generally have the highest CMC, whereas the conjugated form, with the addition of a taurine or glycine reduces the CMC. Interestingly, the addition of more hydrophilic groups to the molecule reduces the CMC (as shown in Figure 1B). This behavior is contradictory to the usual linear surfactants, where a higher hydrophobicity results in a lower CMC [38]. This evident decrease in the CMC with the conjugated BS is due to the stabilization of the micelle due to the hydrogens bonds on the amino residue [39]. The hydrogen bonds between these groups result in added induced dipole interactions of the hydrophobic sections. This has been shown by molecular dynamics simulations [40,41]. Conjugated BS offers additional sites for H-bond formation, especially between the peptide amino group and the steroid hydroxyl groups. Hydrogen bonds were found to be missing in unconjugated primary BS. In addition, the flipped molecules might serve to further stabilize the aggregate by offering more sites for hydrogen bond formation [3]. Comparing primary to secondary BS, dihydroxy secondary unconjugated BS create micelles in a smaller concentration than trihydroxy primary unconjugated BS [42], which follows the usually expected influence of hydrophobicity. After primary micelles are formed, the micelles may further aggregate to form secondary micelles, which are held together by hydrogen bonds [43,44].

The primary unconjugated CMCs had the largest standard deviation (σ = 2.33) because it is the most frequently measured (N = 28) and thus was determined with the largest variety of methods (conductivity, fluorescence, light-scattering, potentiometry, and surface tension), whereas secondary unconjugated BS had the lowest standard deviation (σ = 0.68) since it was determined with a lower variety of methods (tensiometry, conductivity, light-scattering, and conductometry).

In addition, the CMC was shown to change over the years of publication, as shown in Figure S2, where it is evident that the CMC of the measured BS actually increases with time, specifically for the BS sodium deoxycholate increased from ~3.92 mM in the 1960s to 1970s to an average of ~4.16 mM from 2010 to 2020. The only reasonable justification is the increased purity of the tested BSs, which removed a synergistic contaminant.

**Figure 2:**
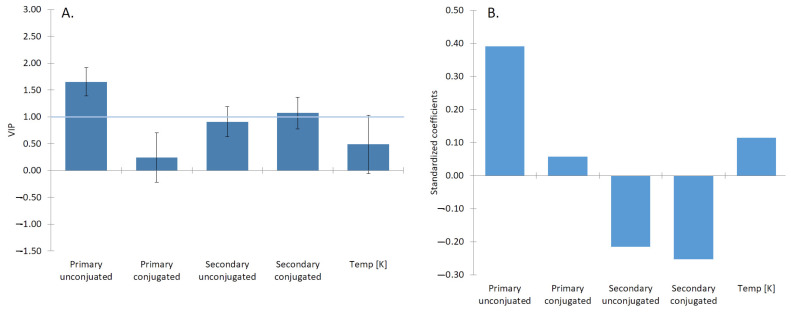
(**A**) The most influential parameters (with VIP larger than 1) affecting the CMC are the type of the BS, specifically the primary unconjugated and the secondary conjugated BS. (**B**) The primary unconjugated BS: cholic and chenodeoxycholic acids showed a positive impact on the creation of the micelles. The location of the OH group at positions 3α and 7α of NaCDC and 3α, 7α, and 12α of NaC promote micellar growth. The same orientation of the OH groups enhances the micelle formation. The location of the OH group and its position can be recognized as the most influential factor promoting micelle formation [3].

These results clearly indicate that the level of conjugation is a significant factor influencing the properties of the BS (as shown in Figure 2). However, it should be noted that BS do not exist as pure compounds in the human body, but as a mixture of primary/secondary and conjugated/unconjugated. Therefore, the interactions of BS in these mixtures still need to be understood.

### 2.2. Analysis of β Parameter

**Figure 3:**
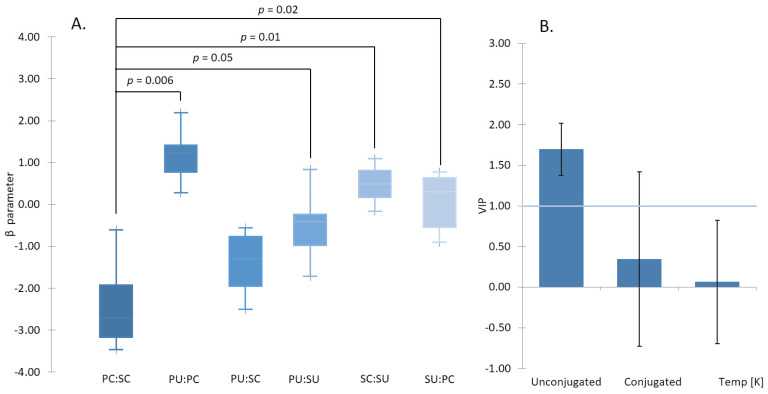
(**A**) The plot of β parameter for different BS:BS mixed systems. A positive β indicates the antagonistic effect and thus a higher CMC, and a negative β indicates a synergistic effect, and thus a lower CMC. The system composed of two conjugated forms of the BS showed the most synergism. The graph was created using six different BS systems. The system composed only of conjugated forms of the BS (PC:SC) showed to be statistically different from almost all other investigated systems, composed of at least one unconjugated form of the BS. (**B**) The most significant factor affecting the BS:BS mixed system was the secondary conjugated form of the BS. The synergistic effect was enhanced in the systems composed of the secondary conjugated aggregates. The *p*-value of PC:SC and SU:PC were calculated to be 0.02.

The interactions of surfactants are characterized by β, as described by Rosen [46]. The beta values were taken from three publications, looking at the synergism or antagonism of mixed micelles of BS systems. All BS were categorized into primary conjugated/unconjugated, and secondary conjugated/unconjugated. The effect of conjugation is shown in Figure 2. However, to determine the contribution of each of the factors (conjugation/deconjugation) to the β, the data were analyzed by partial least squares (PLS) regression, which allowed us to extract the variable importance, as shown in Figure 3. The various categories were included as “one hot encoded” variables. Temperature was included as the degree of counter-ion binding changes with temperature [47].

It can be seen that conjugated secondary BS has the strongest contribution to the β value of mixed BS micelles. The effect is negative, which means a synergistic effect reduces the CMC. Both the primary conjugated, as well as primary unconjugated, have small antagonistic effects. This synergism is due to the lack of the additional hydroxyl group, making the molecule more hydrophobic and enhancing its insertion into the micelle, while the conjugated chain allows for more hydrogen bonds with other molecules, thereby stabilizing the molecule inside the micelle. This can be seen when comparing the number of hydrogen bonds with water, where both GDCH and TDCH have on average 14 hydrogen bonds, compared to 16.5 for conjugated primary BS. The number of hydrogen bonds between conjugated primary and secondary BS is the same.

It clearly shows that conjugation, especially in conjunction with secondary conjugated BS is an important synergistic factor enhancing the micellization of the BS.

When comparing the β values of mixtures of traditional linear surfactants and bile salts, mixtures of linear surfactants generally have synergistic effects due to better packing of a variety of tails lengths into the core of the micelle [7]. This synergistic effect is evident also in the BS mixtures of the same type (conjugated/conjugated and unconjugated/unconjugated), as it can be observed in Figure 3A, and Table 2. However, interestingly mixtures of conjugated/unconjugated (e.g., PC:SU or PU:PC) exhibit an antagonistic effect. This is the result of the columbic repulsion between the negative charge of the carboxyl group of the unconjugated BS with the slightly electro-negative ester of the amino acid. The action of BSH and formation of unconjugated BS, therefore, reduces the ability of the bile to form micelles.

### 2.3. Aggregation Number

**Figure 4:**
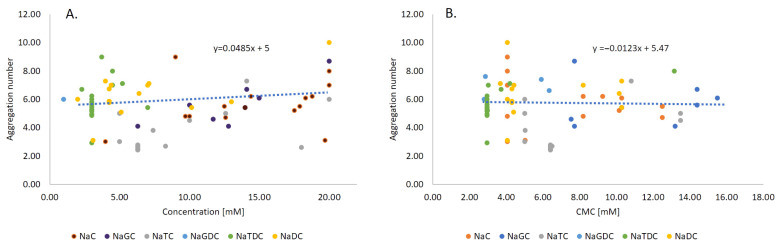
(**A**) Increasing concentration of the BS increases the aggregation number. The lowest aggregation number was seen in primary conjugated BS. (**B**) The data of aggregation number were collected via meta-analysis, and for CMC, we used the averaged CMC from our meta-analysis at temperature range 283.15–323.15 K. The results showed that the aggregation number does not decrease with increasing CMC, which is in contradiction to Madenci et al. [3]. The lack of CMC dependence of the aggregation number is due to the H-bond interaction between the BS molecules and the formation of secondary micelles. The aggregation number of classical surfactants, however, depends on CMC, where the aggregate number increases with increasing CMC [52].

**Figure 5:**
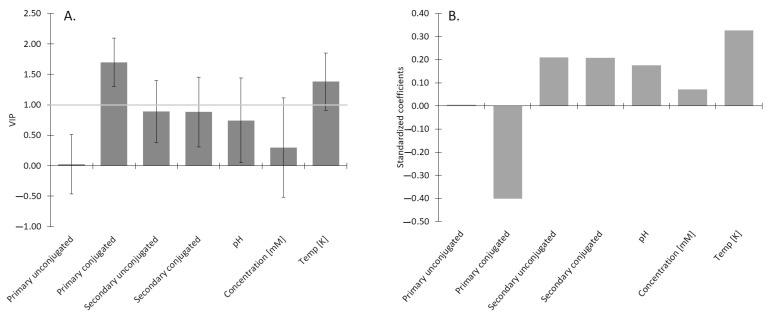
(**A**) The most influential factors influencing the aggregation number are types of the BS, and the temperature. CMC of the BS tends to decrease with increasing temperature up to 303 K, beyond which the CMC starts to increase, leading to an increasing aggregation number [47]. (**B**) Conjugated forms of the BS have a tendency to have lower CMC than their unconjugated forms, therefore, the aggregate number for the conjugated BS should be smaller than for unconjugated ones. The primary conjugated BS showed a high negative correlation towards aggregation number. Conjugated forms of the BS are stabilized not only by the hydrophobic interaction but also by the hydrogen bonding, which means that they require fewer molecules than their unconjugated forms. The positive relation of the concentration [mM] of BS towards aggregation number yields the relation that with increasing BS concentration, the number of the incorporated molecules will increase [42].

Aggregation number indicates the number of the molecules present in the individual micelle created by the surfactant. To analyze the effect of conjugation on the aggregate number, we have collated both experimental (11 papers with 64 datapoints) and molecular dynamics calculations (4 papers with 14 datapoints). The aggregation number of the individual BS can be observed on the Figure 4A,B; raw data is provided in Table S2. The aggregate number is a crucial parameter considering the amount of surfactant incorporated in the aggregate. Factor influencing aggregation number at 303 K are shown in Figure 5. 

Bile salts may form both primary and secondary micelles, as originally stated by Small and Kawamura [43,44]. Primary aggregates which are spherical or slightly oblate in shape, are created by the hydrophobic interaction [53,54], and those aggregates may interact with hydrogen bonds when linked together by the outwardly directed hydrophilic part of the ion constituents, result in the formation of various, complex shapes of secondary micelles such as flattened or rod-like objects known as secondary aggregates [40]. The creation process of the primary and secondary micelles was confirmed by the molecular dynamics simulations [40,42,55,56]. The mechanism of the formation of the micelles was noticed to be different for deoxycholate (di-hydroxy BS) and cholate (tri-hydroxy BS) [40] specifically a micelle created by the cholate remains a dimer even at 30 mM, which are linked by H-bonds, while the deoxycholate creates primary micelles by hydrophobic interaction and the secondary micelles by hydrogen bonding [40]. Cholate, due to the presence of the three hydroxyl groups, possessed a more hydrophobic character than the deoxycholate, which is mainly characterized by hydrophilic edge [40]. It should be noted that in the human body, the concentration of the BS in the gallbladder varies between 10–50 mM [57], where BS are not favorable to form the secondary micelles.

**Figure 6:**
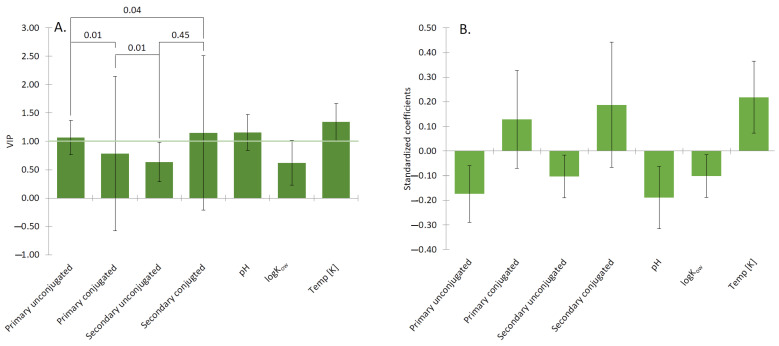
(**A**) The VIP analysis showed that the most significant factors influencing the MSR are the types of the BS, either primary or secondary, and conjugated or unconjugated. (**B**) The standardized coefficient of MSR for different forms of the BS. Elements with the longest bar have the greatest impact on MSR. Their contribution is positive if they are above the line and negative if they are below the line. Statistical significance was determined by using a *t*-test. The MSR of conjugated forms (PC, SC) of the BS have shown to be statistically significant to the MSR of unconjugated BS (PU and SU).

The results from the PLS regression, shown in Figure 6, represent the contribution of different parameters to the aggregation number. The VIP indicated that the type of the BS was the most meaningful parameter affecting the aggregation number. For the conditions of the experiments, the temperature had a higher significance than pH since temperature predominately affects the formation of large aggregates (N_a_ ≥ 10). The increasing temperature balances two opposite effects: the repulsion between anionic polar heads and hydrophobic interaction, ensuring the stability of the small aggregates (primary micelles N_a_ < 10). It has previously been shown that increasing temperature decreases the size of secondary BS micelles [58], which is due to the structure of the secondary aggregates, where hydroxyl groups are hidden inside the micelle and the anionic amino acid residue predominates on the outside of the micelle. pH was shown to have the positive contribution towards the aggregation number since acidification of sodium glycodeoxycholic acid promotes the formation of the helical aggregates [58,59]. Additionally, increasing pH may result in dehydration of nonionic moiety and the formation of hydrogen bonding between nonionic polar parts, allowing larger micelles to form [60].

### 2.4. Molar Solubilization Ratio

The molar solubilization ratio is known as the ratio of the molecules solubilized inside the aggregate. For bile salts, the MSR has a crucial meaning, as it is the size of the MSR that will dictate the efficiency of removal of the lipolysis products from the lipid droplet.

We may observe in Figure 6B that the logK_ow_ has a negative contribution to the MSR, which is the result of the correlation between the logK_ow_ of the solubilizate and its molecular volume (Pearson correlation for the 21 solubilizates analyzed here was 0.990, *p* < 0.0001). Therefore, more hydrophobic solubilizates in our analysis resulted in a lower MSR due to their larger volume. The meta-analysis of six papers for the first time highlights the effect of conjugation on the MSR. Both primary and secondary unconjugated BS have a negative contribution to the MSR. That is, the higher is the level of the unconjugated forms of the BS the lower will be MSR for a given substance. In addition, the MSR is also influenced by the locus of solubilization within the micelle. Steroid compounds were found to be more effectively incorporated into the NaDC than NaC due to the less hydrophilic character of the NaC molecule [61]. Aromatic compounds not only have the ability to solubilize inside the hydrophobic interior, but also occupy external positions on BS micelles, as determined by Kolehmainen et al. [62]. Unconjugated BS was more favorable to incorporate fatty acids into their structure than their glycine conjugates [63]. Therefore, vitamins undergo lipolysis before they are absorbed by the BS micelles.

### 2.5. Measurements of Interfacial Tension at Oil/BS Interface

**Figure 7:**
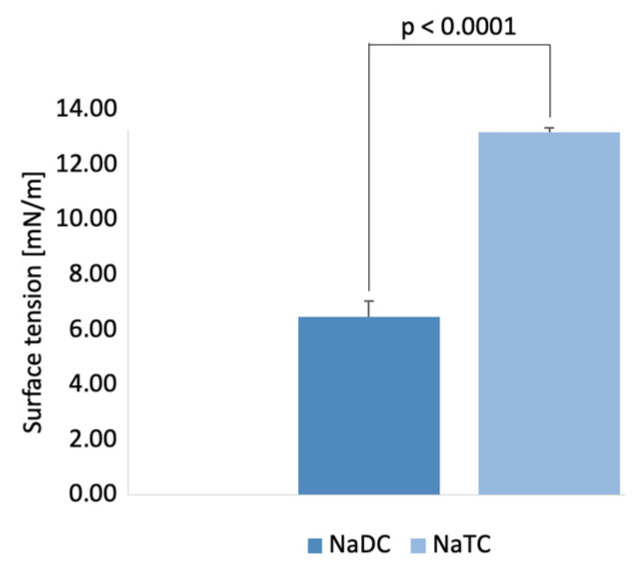
The surface tension of sunflower oil droplets with 10 mM of the two BS, NaDC and NaTC, measured at 310.15 K. Surface tension of PC NaTC showed to be statistically significant lower compared to the SU NaDC. The average surface tension was determined to be 6.45 ± 0.01 for NaDC and 13.17 ± 0.08 for NaTC. The control sunflower oil droplet had a surface tension of 30.64 ± 0.13 mN/m.

The ability of the two different BS NaTC and NaDC to decrease the surface tension of the oil droplets was investigated to determine their role in the lipolysis process. Surface tension reduction of BS follows a similar trend as shown by the CMC. A higher CMC of NaTC (Figure 1A) indicates a lower ability of surface tension reduction of the oil droplet at physiological conditions (Figure 7). NaDC showed a greater ability to reduce the oil droplets’ surface tension and may therefore better reduce the droplet size during the lipolysis process.

### 2.6. Impact of Conjugation Form of the BS on the Lipid Digestion

**Figure 8:**
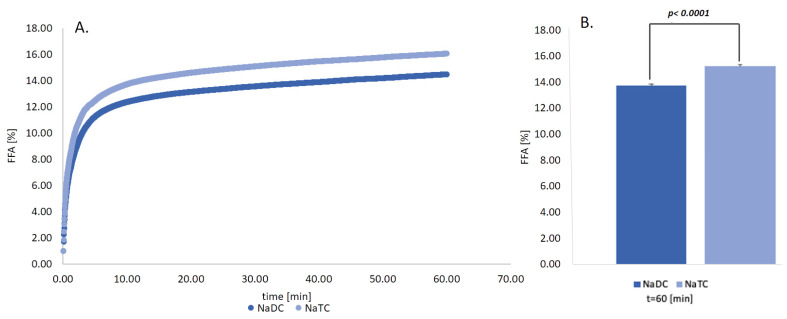
(**A**) FFA released over time from WPI-stabilized emulsion in respect to two different forms of BS: NaTC and NaDC at 10mM under physiological conditions at 310.15 K. Experimental results of CMC of NaTC and NaDC at 310.15 K with errors are also shown. The CMC of NaTC and NaDC have been shown to be statistically significant, *p* < 0.0001, and are comparable to previously determined values (5–20% of FFA after 60 min for NaTC and NaTDC at 10 mM and 50 mM, methylcellulose stabilized emulsion [64], 10–13% of FFA after 60 min for mix BS at 9.7 mM, WPI-stabilized emulsion [65], 6.5–15% of FFA after 60 min. for mix BS at 10 mM [66]. (**B**) Statistical significance was calculated by using the t-sample t-test. The magnitude of the %FFA is in line with results from other researchers who also digested sunflower oil/WPI, or other protein emulsions, with similar emulsion sizes [64,65,67]. *p* < 0.0001 indicates that the values are statistically significant.

To show that the changes in the proposed surface parameters actually affect the rate of lipolysis as hypothesized above, we have conducted experiments to show the significance of the conjugation of the BS. In essence, the rate of lipolysis was determined in in-vitro experiments with both PC and SU bile salts. The results are shown in Figure 8. It is shown that conjugated NaTC shows a faster rate of release of FFA (i.e., lipolysis) as compared to the same concentration of the unconjugated counterpart NaDC.

**Figure 9:**
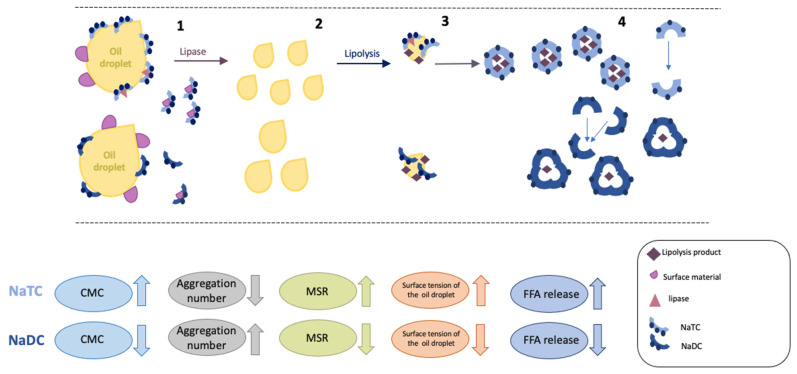
The role of BS during the lipolysis process. 1. Bile salt adsorbs at the oil droplet, leading to the removal of surface materials (proteins, emulsifiers) and promote adsorption of the lipase. 2. BSs break down larger lipid droplets into smaller ones ensuring efficient lipolysis 3. BS will assist lipase and co-lipase in the sorption onto the emulsion. 4. BS incorporates lipolysis products into mixed micelles, ensuring the transport of valuable components into the body.

Previously it was pointed out that the interfacial process of lipolysis involves three key steps [68]. However, we are showing that the release of the FFA from the emulsion (as shown schematically in Figure 9) is linked to five factors. First, the ability of the BS to further break down the emulsified lipid droplets, which promotes a larger surface area onto which the enzyme can adsorb [9]. Second, the adsorption kinetics of the BS onto the emulsion. Third, assisting the lipase/co-lipase complex to attach to the emulsion surface. Fourth, is the removal of lipolysis products from the oil/water interface, and finally, the desorption kinetics from the emulsion [65,69].

In our case, it could be observed that the conjugated form of the BS, NaTC, favored the release of FFA to a higher extend than SU form of the BS. From the five factors mentioned above, we hypothesize from Figure 6 that the enhanced MSR of the primary conjugated aids the removal of the lipolysis products from the emulsion, with a small number of BS molecules in the micelle (Figure 5), in addition, the added BS–water hydrogens bonds (as shown in Table 3) allow the primary conjugated BS to desorb easier from the surface of the emulsion. Thus, the lower CMC of NaDC (with less BS–water hydrogens bonds) (Figure 1) would yield a lower release of FFA (Figure 5) as in comparison to NaTC [70]. Interestingly, as shown in Figure 7, the interface activity of the unconjugated BS is higher than the conjugated, which therefore indicates that lipolysis is dominated by other factors. BS have previously been shown to have different ability to adsorb at the oil–water interface [64,71], and thus to reduce the interfacial tension of the droplet. Micellar state affects the BS adsorption [72]. NaDC showed a better ability to reduce interfacial tension on the adsorbed droplet (Figure 7), thus promoting a greater surface area for lipase and co-lipase adsorption. However, accumulated products on the emulsion interface and the lower ability of SU BS to remove surface materials may interrupt adsorption of the lipase and co-lipase and, therefore, can result in lower FFA release for SU BS. In the future, more detailed adsorption/desorption studies of BS behavior at oil interface should be performed to better understand their role in the lipolysis process, as well as to determine which of the five factors dominate the lipolysis process.

In summary, the significantly lower release of FFA obtained by NaDC indicates that deconjugation of the BS affects lipid metabolism. It, therefore, follows that excessive microbiota with BSH may impact on the efficiency of lipid digestion. Moreover, the reduction of digestion performance by NaDC suggests that the bacterial action and composition of gut microflora in our organism have an important impact on our health and are therefore indirect factors regulating the lipolysis process.

## 3. Materials and Methods

### 3.1. Meta-Analysis

To analyze the importance of BS conjugation, a meta-analysis was performed on experimental data that analyzed the critical micelle concentration (CMC), molar solubilization ratio (MSR), and aggregate numbers of bile salts. This was collected from scientific articles ranging from 1962 to 2019, where Google Scholar was used [73] with the following keywords for CMC: “bile salts, critical micelle concentration, mixed micelle”; for aggregation number: “bile salts, aggregation number”; for MSR: “bile salts, molar solubilization ratio”. This has resulted in 205 unique datapoints of CMC of pure compounds from 27 publications, in 33 datapoints of CMC of mixed systems from 3 publications, 166 datapoints of aggregation number from 9 publications, and 53 datapoints of MSR from 5 publications.

All units were standardized. Additional parameters were noted and included; for CMC temperature and method of determination, for aggregate number temperature, the concentration of BS, CMC, pH and salt concentration, and for MSR, the solubilizate, and temperature, and the salt concentration were noted. The logK_ow_ and molecular volume (nm^3^) of the solubilizate were determined using Molinspiration Cheminformatics. Direct comparisons were only made for systems of the same temperature unless stated otherwise. β and MSR, if not presented, were calculated using the CMC values provided by each author.

### 3.2. Critical Micelle Concentration Determination

The CMC of NaDC (from Sigma Aldrich, St. Louis, MO, USA, 97.0%) and NaTC (from Sigma Aldrich; 97.0%) at physiological temperature (310.15 K) were assessed by using conductivity measurements using an auto titrator (Cerko Lab System CLS/M/07/06, Gdynia, Poland) equipped with a microconductivity electrode (Eurosensor, EPST-2ZAM, Gliwice, Poland). The temperature was maintained using a thermostatic water bath (PolyScience 9106, Niles, IL, USA). The breakpoint determination in the conductivity curves was done using the Phillips method as previously described by Łuczak et al. [74]. The data are given in the Supplementary Materials, Table S1.

### 3.3. Emulsion

Oil in water (O/W) emulsion (oil to water 20:75% *w/w*) and whey protein isolate (WPI) concentration of 0.5% (*w/w*) was prepared by dissolving WPI in saline buffer (150 mM NaCl and 0.02% *w/w* NaN_3_). The mixture was stirred with a magnetic stirrer until dissolution. Sunflower oil, which was previously treated with florisil (Taufkirchen, Sigma, F9127), was used as the oil phase [75]. The mixture of sunflower oil and protein dispersion was further vortexed for 3 min to obtain a coarse emulsion. The pre-emulsion was sonicated with an ultrasound generator (Sonics VCX 500, Sonics & Materials Inc., Newtown, CT, USA) with a 0.13 cm diameter titanium probe with an amplitude of 80%, pulse duration of 5 s on/10 s off for 3 min. Lipolysis results were carried out on split samples, one-half for each bile salt.

### 3.4. Droplet Size

A zetasizer (Zetasizer Nano, Malvern Instruments Ltd., Malvern, UK) was used to determine mean droplet diameter by using dynamic light scattering. Water was used as a dispersant (refractive index of 1.330). The absorbance value of the oil droplets was 0.001 (refractive index of 1.467) [76]. The results of particle size were recorded as the Z-average mean diameter, which is calculated from the particle size distribution [77]. The 2 μL emulsions were diluted in 7 mL of the saline buffer to avoid back-scattering. Each sample was measured in quadruplicate. Exemplary particle size distribution is provided in the Figure S1

### 3.5. Interfacial Tension Measurements

Drop shape analysis was done by measuring interfacial tension using a drop shape analyzer (Krϋss Drop shape analyzer DSA 10, Hamburg, Germany). The measurements were performed as described previously by Szumała et al. [75] with some modifications. Specifically, the measuring cell was filled with 10 mM BS. Subsequently, the oil drop was formed and BS adsorbed on the oil/water interface and interfacial tension was measured. Each drop was allowed to equilibrate with the BS for 10 min before the surface tension was recorded. All measurements were made at 310.15 K, with five repetitions.

### 3.6. In Vitro Duodenal Digestion

In-vitro lipolysis [78] was used to simulate the environmental condition of the small intestine (duodenum). 0.8 mL of the simulated intestinal fluid and 0.375 mL of the emulsion were added to the vessel. After gently mixing with a magnetic stirrer (1500 rpm), 0.3 mL of 10 mM BS (NaTC or NaDC) and 3 μL of 0.3 M CaCl_2_ were pipetted, and the pH was set to 7.0 using 0.1 M HCl. Finally, with the addition of 1.0 mL of freshly prepared pancreatin (80 U/mg of oil) the titration was started.

The reaction vessel was continuously stirred and thermostatically controlled to maintain 310.15 K. All lipolysis experiments were carried out in triplicate.

The extent of the lipolysis was measured by continuous titration with an autotitrator (Cerko Lab System CLS/M/07/06, Gdynia, Poland) of free fatty acids (FFA) with 0.1 M NaOH.

### 3.7. Statistical Analysis

**Figure 10:**
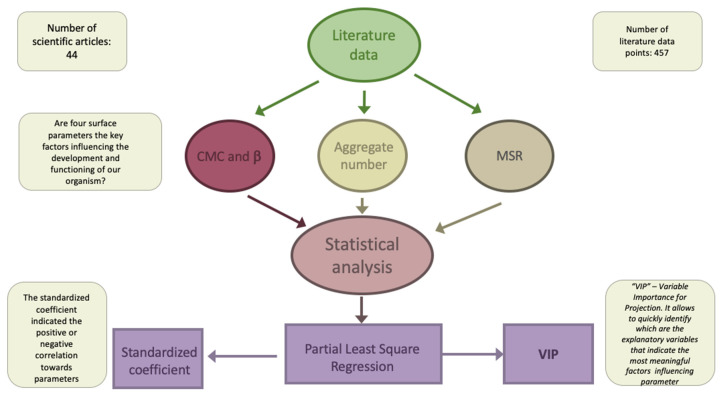
The workflow of the data analysis.

PLS was applied to determine the most significant influence of the descriptor on the dependent variable. The PLS can be used for qualitative as well as for quantitative data, therefore, the PLS analysis was done according to Łozińska et al. [79], and *p*-values were determined by (one-tailed) students t-test. The aim of the analysis of the data was to investigate the potential of which different descriptors influence the specific parameter (CMC, β, N_a_, and MSR). Therefore, we were looking to which extent each descriptor impact on the parameter. The complete data is given in the Supplementary Materials, as Table S1 for the CMC data, Table S2 for β values, Table S3 for aggregation numbers, and Table S4 for the MSR. Statistical analysis was done using XLSTAT (version 2020.1.3.65326) [80]. The workflow for the analysis is schematically represented in Figure 10. Statistical significance is shown in Figures if *p* was determined to be less than 0.05.

## 4. Conclusions

To assess the importance of the level of conjugation, this paper represents the meta-analysis of four phenomenological parameters (CMC, β, N_a_, and MSR).

The conjugated BS will form micelles with a lower concentration than their unconjugated forms. It was shown by molecular dynamic simulations [42,44,56,57] that the lower CMC of the conjugated BS is a result of the hydrogen bonds on the amino acid residues [39]. Secondary conjugated BS showed the greatest contribution to promote the synergistic effect in combination with other BS. Conjugated BS requires fewer molecules to create aggregates, which means that with the same amount of substance, the conjugated BS will promote the formation of more micelles than their unconjugated forms [3]. Although the deconjugation process promoted by BSH will lead to decreasing the CMC of the existing BS, mixed BS systems composed of unconjugated forms of the BS will be characterized by an antagonistic effect, resulting in a higher CMC of the mixed system [48]. Finally, fewer compounds would be solubilizing inside the micelle of the unconjugated BS, which may promote the deficiency of the beneficial compounds in our organism, such as vitamins, fats, and sterols [81].

To prove the importance of conjugation, we have measured the in-vitro digestion of an emulsion with both conjugated and unconjugated bile salts, and we show for the first time experimentally that these changes in lipolysis can be modulated by variation of BS conjugation level. That means that an exemplary decrease in BSH activity (by taking antibiotics, for example) may lead to a potential increase in conjugation, and thus an increase in lipolysis and could cause obesity over a longer period [82], which will, in turn, result in bile saturation [83] and can lead to gallstone formation [84]. On the other hand, overactivity of BSH will result in lowering the level of conjugated BS, which binds strongly to the FXR to reduce bile acid synthesis and result in malnutrition.

## Figures and Tables

**Table 1:** BS composition in the small intestine. Composition of the BS in the small intestine in respect to the concentration of their conjugated and unconjugated forms. The BS appears in its conjugated form in the duodenum and jejunum; the deconjugation process is mostly observed in the ileum.

Duodenum			Jejunum			Ileum		
Conjugated [%]	Unconjugated [%]	Ref.	Conjugated [%]	Unconjugated [%]	Ref.	Conjugated [%]	Unconjugated [%]	Ref.
99.70	0.30	[35]	100.00	0.00	[35]	88.00	11.79	[36]
94.20	5.00	[35]	96.50	3.50	[35]	75.00	25.00	[37]
91.00	9.00	[37]	84.00	15.50	[37]			

**Table 2:** The binary mixtures of the cationic/cationic, anionic/anionic and BS/BS surfactants at 298.15 K. The common linear surfactants showed a synergistic effect, while BS yielded both synergistic and antagonistic effect. Exemplary data for BS is given, where a complete BS mixture data set is given in Table S3.

Type of Surfactant	Composition	CMC [mM]	β	References
NaC/NaTC	0.2	6.1	1.33	[48]
(PU:PC)	0.4 0.6 0.8	8.1 9.18 9.93	2.19 1.39 1.48	[48]
NaC/NaDC	0.2	3.6	–0.40	[48]
(PU:SU)	0.4 0.6 0.8	4.15 4.8 5.41	−0.31 −0.41 −0.84	[48]
C_12_TAB/C_10_TAB	0.3	25.00	−1.4	[49]
C_14_TAB/C_10_TAB	0.3	8.00	−4.7	[49]
C_14_TAB/C_12_TAB	0.3	6.00	−1.4	[49]
C_16_TAB/C_10_TAB	0.3	3.00	−7.7	[49]
C_16_TAB/C_12_TAB	0.3	3.00	−5.1	[49]
C_16_TAB/C_14_TAB	0.3	2.00	−1.5	[49]
C_16_Br/C_16_BzCl	0.10 0.25 0.5 0.75 0.90	13.20 9.33 10.70 22.90 24.10	−4.24 −4.83 −2.95 −1.27 −1.79	[50]
SOS/SAE2S			−4.98	[51]

**Table 3:** The number of BS-BS hydrogen bonds and number of BS–water hydrogen bonds of four groups of BS [41]. Secondary unconjugated BS could be characterized by the smallest number of BS-BS HBs and number of BS–water HBs, while the primary conjugated has the highest number of BS–water HBs.

Molecule	No. of BS–BS HBs	No. BS–Water HBs
Primary unconjugated	0.08 ± 0.13	15.00 ± 1.41
Primary conjugated	0.305 ± 0.35	16.50 ± 1.41
Secondary unconjugated	0.06 ± 0.11	13.00 ± 1.41
Secondary conjugated	0.305 ± 0.30	14.00 ± 1.41

## Data Availability

Provided in the Supplementary Materials.

## Supplementary Materials

The following are available online, Table S1: CMC data, Table S2: β values of binary mixtures of bile salts, Table S3: Aggregation numbers of BS, Table S4: MSR, Figure S1: Particle size distribution of O/W emulsion, and Figure S2: CMC of NaDC change with years.

## Sample Availability

Not available.
