# Supplementary material for: Importance of Conjugation of the Bile Salt on the Mechanism of Lipolysis
Source: Molecules. 2021 Sep 23;26(19):5764. doi: 10.3390/molecules26195764 (PMC8510408; doi:10.3390/molecules26195764)
Supplement: Supplementary file 1 [file molecules-26-05764-s001.zip › molecules-1347531-supplementary.pdf]

Importance of conjugation of the bile salt on the mechanism of lipolysis

Natalia Łozińska, and Christian Jungnickel*

Department of Colloid and Lipid Science, Faculty of Chemistry, Gdańsk University of Technology

ul. Narutowicza 11/12, Gdańsk 80-233, Poland,

*Corresponding author: Christian Jungnickel, email:christian.jungnickel@pg.edu.pl

Supporting Information

Table S1 CMC data

Bile salt	CMC	Temperature [K]	Reference
NaC	10.80	283.15	(Mukherjee et al. 2016)
NaC	12.50	283.15	(Mukherjee et al. 2016)
NaC	17.90	283.15	(Olesen, Westh, and Holm 2015)
NaC	8.80	283.15	(Maestre, Guardado, and Moyá 2014)
NaC	8.02	293.15	(Rub et al. 2013)
NaC	7.78	293.15	(Mukherjee et al. 2016)
NaC	8.25	293.15	(Mukherjee et al. 2016)
NaC	8.02	293.15	(Kabir-ud-Din, Rub, and Naqvi 2011)
NaC	14.40	293.15	(Kumar, Patial, and Chauhan 2015)
NaC	8.67	298.15	(Rub et al. 2013)
NaC	9.70	298.15	(Olesen et al. 2015)
NaC	9.00	298.15	(Azum, Rub, and Asiri 2019)
NaC	9.00	298.15	(Miyajima, K., Yokoi, M., Komatsu, H., & Nakagaki 1986)
NaC	10.00	298.15	(Faustino et al. 2014)
NaC	11.30	298.15	(Faustino et al. 2014)
NaC	6.20	298.15	(Ninomiya, Matsuoka, and Moroi 2003)
NaC	12.90	298.15	(Ćirin, Poša, and Krstonošić 2011)
NaC	12.78	298.15	(Ćirin et al. 2011)
NaC	12.90	298.15	(Ćirin et al. 2011)
NaC	12.73	298.15	(Ćirin et al. 2011)
NaC	16.00	298.15	(Subuddhi and Mishra 2007)
NaC	10.20	298.15	(Yadav, Parikh, and Kumar 2017)
NaC	13.00	298.15	(Roda, Hofmann, and Mysels 1983)
NaC	11.10	298.15	(Poša, Ćirin, and Krstonošić 2013)
NaC	8.67	298.15	(Kabir-ud-Din et al. 2011)
NaC	14.10	298.15	(Kumar et al. 2015)
NaC	7.98	298.15	(Mahajan and Mahajan 2012)
NaC	6.20	298.15	(Matsuoka and Moroi 2002)
NaC	11.00	298.15	(Nakashima et al. 2002)
NaC	10.20	298.15	(Maestre et al. 2014)

NaC	9.10	298.15	(Poša et al. 2015)
NaC	8.80	298.15	(Poša et al. 2015)
NaC	5.18	303.15	(Jana and Moulik 1991)
NaC	9.12	303.15	(Jana and Moulik 1991)
NaC	7.22	303.15	(Jana and Moulik 1991)
NaC	9.06	303.15	(Rub et al. 2013)
NaC	5.89	303.15	(Mukherjee et al. 2016)
NaC	7.35	303.15	(Mukherjee et al. 2016)
NaC	9.06	303.15	(Kabir-ud-Din et al. 2011)
NaC	14.00	303.15	(Kumar et al. 2015)
NaC	6.10	310.15	(Mukherjee et al. 2016)
NaC	18.80	310.15	(Olesen et al. 2015)
NaC	7.50	310.15	(Mukherjee et al. 2016)
NaC	9.40	310.15	(Maestre et al. 2014)
NaC	14.30	313.15	(Kumar et al. 2015)
NaC	17.50	323.15	(Olesen et al. 2015)
NaC	19.10	323.15	(Maestre et al. 2014)
NaCDC	3.00	298.15	(Ninomiya et al. 2003)
NaCDC	9.00	298.15	(Roda et al. 1983)
NaCDC	4.60	298.15	(Nakashima et al. 2002)
NaCDC	5.80	298.15	(Poša et al. 2015)
NaCDC	5.50	298.15	(Poša et al. 2015)
NaDC	2.95	283.15	(Sugihara and Tanaka 1976)
NaDC	6.30	283.15	(Olesen et al. 2015)
NaDC	4.65	283.15	(Mukherjee et al. 2016)
NaDC	5.82	283.15	(Mukherjee et al. 2016)
NaDC	6.60	283.15	(Maestre et al. 2014)
NaDC	3.35	283.15	(Sugihara and Tanaka 1976)
NaDC	3.24	283.15	(Sugihara and Tanaka 1976)
NaDC	2.30	283.15	(Matsuoka and Moroi 2002)
NaDC	3.55	293.15	(Sugihara and Tanaka 1976)
NaDC	3.47	293.15	(Sugihara and Tanaka 1976)
NaDC	1.50	293.15	(Carey and Small 1969)
NaDC	2.95	293.15	(Rub et al. 2013)
NaDC	3.80	293.15	(Mukherjee et al. 2016)
NaDC	5.36	293.15	(Mukherjee et al. 2016)
NaDC	2.95	293.15	(Kabir-ud-Din et al. 2011)
NaDC	6.00	293.15	(Kumar et al. 2015)
NaDC	4.10	298.15	(Juna, K., & Sugano 1969)
NaDC	2.00	298.15	(Small 1971)
NaDC	4.30	298.15	(Olesen et al. 2015)
NaDC	7.94	298.15	(Sugihara and Tanaka 1976)
NaDC	1.70	298.15	(Sugihara and Tanaka 1976)
NaDC	4.20	298.15	(Miyajima, K., Yokoi, M., Komatsu, H., & Nakagaki 1986)
NaDC	4.50	298.15	(Jana and Moulik 1991)

NaDC	2.40	298.15	(Matsuoka and Moroi 2002)
NaDC	4.00	298.15	(Nakashima et al. 2002)
NaDC	2.40	298.15	(Ninomiya et al. 2003)
NaDC	6.00	298.15	(Subuddhi and Mishra 2007)
NaDC	4.30	298.15	(Ćirin et al. 2011)
NaDC	4.25	298.15	(Ćirin et al. 2011)
NaDC	4.16	298.15	(Ćirin et al. 2011)
NaDC	3.02	298.15	(Kabir-ud-Din et al. 2011)
NaDC	4.30	298.15	(Ćirin et al. 2011)
NaDC	4.25	298.15	(Ćirin et al. 2011)
NaDC	2.90	298.15	(Mahajan and Mahajan 2012)
NaDC	3.02	298.15	(Kabir-ud-Din et al. 2011)
NaDC	3.07	298.15	(Poša et al. 2013)
NaDC	5.56	298.15	(Faustino et al. 2014)
NaDC	5.74	298.15	(Faustino et al. 2014)
NaDC	4.50	298.15	(Maestre et al. 2014)
NaDC	5.40	298.15	(Kumar et al. 2015)
NaDC	4.50	298.15	(Yadav et al. 2017)
NaDC	3.25	298.15	(Azum et al. 2019)
NaDC	3.97	303.00	(Jana and Moulik 1991)
NaDC	2.75	303.00	(Jana and Moulik 1991)
NaDC	7.94	303.00	(Jana and Moulik 1991)
NaDC	4.80	303.00	(Sugihara and Tanaka 1976)
NaDC	4.57	303.00	(Sugihara and Tanaka 1976)
NaDC	3.11	303.15	(Rub et al. 2013)
NaDC	3.02	303.15	(Mukherjee et al. 2016)
NaDC	4.05	303.15	(Mukherjee et al. 2016)
NaDC	3.11	303.15	(Kabir-ud-Din et al. 2011)
NaDC	5.10	303.15	(Kumar et al. 2015)
NaDC	8.20	310.15	(Maestre et al. 2014)
NaDC	8.12	310.15	
NaDC	8.20	310.15	(Olesen et al. 2015)
NaDC	6.10	313.15	(Sugihara and Tanaka 1976)
NaDC	3.16	313.15	(Mukherjee et al. 2016)
NaDC	4.32	313.15	(Mukherjee et al. 2016)
NaDC	5.90	313.15	(Kumar et al. 2015)
NaDC	10.20	323.15	(Maestre et al. 2014)
NaDC	10.10	323.15	(Olesen et al. 2015)
NaGC	13.60	283.15	(Maestre et al. 2014)
NaGC	12.80	283.15	(Olesen et al. 2015)
NaGC	6.80	298.15	(Maestre et al. 2014)
NaGC	4.20	298.15	(Small 1971)
NaGC	6.30	298.15	(Olesen et al. 2015)
NaGC	7.00	298.15	(Miyajima, K., Yokoi, M., Komatsu, H., & Nakagaki 1986)
NaGC	12.00	298.15	(Roda et al. 1983)

NaGC	10.00	298.15	(Nakashima et al. 2002)
NaGC	14.70	310.15	(Maestre et al. 2014)
NaGC	14.10	310.15	(Olesen et al. 2015)
NaGC	16.00	323.15	(Maestre et al. 2014)
NaGC	15.00	323.15	(Olesen et al. 2015)
NaGCDC	6.00	298.15	(Roda et al. 1983)
NaGCDC	2.30	298.15	(Olesen et al. 2015)
NaGCDC	7.00	298.15	(Roda et al. 1983)
NaGCDC	7.00	298.15	(Roda et al. 1983)
NaGCDC	2.40	298.15	(Nakashima et al. 2002)
NaGCDC	2.10	310.15	(Olesen et al. 2015)
NaGDC	5.80	283.15	(Maestre et al. 2014)
NaGDC	5.60	283.15	(Olesen et al. 2015)
NaGDC	6.00	298.15	(Roda et al. 1983)
NaGDC	3.30	298.15	(Olesen et al. 2015)
NaGDC	2.12	298.15	(Kratohvil and DelliColli 1968)
NaGDC	1.10	298.15	(Small 1971)
NaGDC	1.90	298.15	(Small 1971)
NaGDC	2.20	298.15	(Nakashima et al. 2002)
NaGDC	3.43	298.15	(Maestre et al. 2014)
NaGDC	6.00	310.15	(Maestre et al. 2014)
NaGDC	5.80	310.15	(Olesen et al. 2015)
NaGDC	6.60	323.15	(Maestre et al. 2014)
NaGDC	6.10	323.15	(Olesen et al. 2015)
NaTC	6.79	283.15	(Mukherjee et al. 2016)
NaTC	7.92	283.15	(Mukherjee et al. 2016)
NaTC	4.00	283.15	(Rub et al. 2014)
NaTC	8.30	283.15	(Olesen et al. 2015)
NaTC	3.20	283.15	(Carey and Small 1969)
NaTC	8.80	283.15	(Maestre et al. 2014)
NaTC	2.80	293.15	(Carey and Small 1969)
NaTC	4.25	293.15	(Kabir-ud-Din et al. 2011)
NaTC	4.25	293.15	(Rub et al. 2013)
NaTC	6.68	293.15	(Mukherjee et al. 2016)
NaTC	7.30	293.15	(Mukherjee et al. 2016)
NaTC	4.70	298.15	(Roda et al. 1983)
NaTC	4.50	298.15	(Rub et al. 2013)
NaTC	4.50	298.15	(Kabir-ud-Din et al. 2011)
NaTC	5.00	298.15	(Olesen et al. 2015)
NaTC	4.70	298.15	(Azum et al. 2019)
NaTC	6.00	298.15	(Miyajima, K., Yokoi, M., Komatsu, H., & Nakagaki 1986)
NaTC	5.60	298.15	(Maestre et al. 2014)
NaTC	4.75	303.15	(Rub et al. 2013)
NaTC	4.75	303.15	(Kabir-ud-Din et al. 2011)
NaTC	6.14	303.15	(Mukherjee et al. 2016)

NaTC	6.81	303.15	(Mukherjee et al. 2016)
NaTC	3.10	303.15	(Carey and Small 1969)
NaTC	13.70	310.15	(Maestre et al. 2014)
NaTC	12.60	310.15	(Olesen et al. 2015)
NaTC	12.99	310.15	
NaTC	6.36	313.15	(Mukherjee et al. 2016)
NaTC	7.20	313.15	(Mukherjee et al. 2016)
NaTC	3.00	313.15	(Carey and Small 1969)
NaTC	3.30	323.15	(Carey and Small 1969)
NaTC	14.10	323.15	(Olesen et al. 2015)
NaTC	15.00	323.15	(Maestre et al. 2014)
NaTDC	2.01	283.15	(Mukherjee et al. 2016)
NaTDC	2.21	283.15	(Mukherjee et al. 2016)
NaTDC	4.50	283.15	(Olesen et al. 2015)
NaTDC	1.80	283.15	(Carey and Small 1969)
NaTDC	4.56	283.15	(Maestre et al. 2014)
NaTDC	1.88	293.15	(Mukherjee et al. 2016)
NaTDC	2.62	293.15	(Mukherjee et al. 2016)
NaTDC	1.50	293.15	(Carey and Small 1969)
NaTDC	6.00	298.15	(Jana and Moulik 1991)
NaTDC	3.05	298.15	(Kratohvil and DelliColli 1968)
NaTDC	2.87	298.15	(Kratohvil and DelliColli 1968)
NaTDC	2.30	298.15	(Olesen et al. 2015)
NaTDC	4.07	298.15	(Roda et al. 1983)
NaTDC	4.00	298.15	(Kratohvil, Hsu, and Kwok 1986)
NaTDC	2.30	303.15	(Jana and Moulik 1991)
NaTDC	3.98	303.15	(Jana and Moulik 1991)
NaTDC	4.07	303.15	(Jana and Moulik 1991)
NaTDC	2.43	303.15	(Mukherjee et al. 2016)
NaTDC	2.90	303.15	(Mukherjee et al. 2016)
NaTDC	1.80	303.15	(Carey and Small 1969)
NaTDC	2.88	313.15	(Mukherjee et al. 2016)
NaTDC	3.50	313.15	(Mukherjee et al. 2016)
NaTDC	2.10	313.15	(Carey and Small 1969)
NaTDC	4.53	310.15	(Maestre et al. 2014)
NaTDC	4.50	310.15	(Olesen et al. 2015)
NaTDC	2.10	323.15	(Carey and Small 1969)
NaTDC	5.30	323.15	(Maestre et al. 2014)
NaTDC	5.20	323.15	(Olesen et al. 2015)
TCDC	2.30	298.15	(Olesen et al. 2015)
TCDC	7.00	298.15	(Roda et al. 1983)
TCDC	2.10	310.15	(Olesen et al. 2015)

References for Table S1

Azum, Naved, Malik Abdul Rub, and Abdullah M. Asiri. 2019. "Bile Salt–Bile Salt Interaction in

Mixed Monolayer and Mixed Micelle Formation." Journal of Chemical Thermodynamics 128:406–14.

Carey, Martin C. and Donald M. Small. 1969. "Micellar Properties of Dihydroxy and Trihydroxy Bile Salts: Effects of Counterion and Temperature." Journal of Colloid And Interface Science 31(3):382–96.

Ćirin, Dejan M., Mihalj M. Poša, and Veljko S. Krstonošić. 2011. "Interactions between Selected Bile Salts and Triton X-100 or Sodium Lauryl Ether Sulfate." Chemistry Central Journal 5(1):89.

Faustino, Célia M. C., Cláudia S. Serafim, Inês N. Ferreira, Mafalda A. Branco, António R. T. Calado, and Luis Garcia-Rio. 2014. "Mixed Micelle Formation between an Amino Acid-Based Anionic Gemini Surfactant and Bile Salts." Industrial and Engineering Chemistry Research 53(24):10112–18.

Jana, Pijush Kanti and Satya Priya Moulik. 1991. "Interaction of Bile Salts with Hexadecyltrimethylammonium Bromide and Sodium Dodecyl Sulfate." Journal of Physical Chemistry 95(23):9525–32.

Juna, K., & Sugano, T. 1969. "Light Scattering by Aqueous Solutions of Sodium Cholate." Nippon Kagaku Zasshi 90:463-466.

Kabir-ud-Din, Malik Abdul Rub, and Andleeb Z. Naqvi. 2011. "Aqueous Amphiphilic Drug (Amitriptyline Hydrochloride)-Bile Salt Mixtures at Different Temperatures." Colloids and Surfaces B: Biointerfaces 84(2):285–91.

Kratohvil, J. P. and H. T. DelliColli. 1968. "Micellar Properties of Bile Salts. Sodium Taurodeoxycholate and Sodium Glycodeoxycholate." Canadian Journal of Biochemistry 46(8):945–52.

Kratohvil, Josip P., Wan P. Hsu, and Daw I. Kwok. 1986. "How Large Are the Micelles of Di-α-Hydroxy Bile Salts at the Critical Micellization Concentrations in Aqueous Electrolyte Solutions? Results for Sodium Taurodeoxycholate and Sodium Deoxycholate." Langmuir 2(2):256–58.

Kumar, Kuldeep, Baljeet S. Patial, and Suvarcha Chauhan. 2015. "Conductivity and Fluorescence Studies on the Micellization Properties of Sodium Cholate and Sodium Deoxycholate in Aqueous Medium at Different Temperatures: Effect of Selected Amino Acids." Journal of Chemical Thermodynamics 82:25–33.

Maestre, Alfredo, Pilar Guardado, and María Luisa Moyá. 2014. "Thermodynamic Study of Bile Salts Micellization." Journal of Chemical and Engineering Data 59(2):433–38.

Mahajan, Suruchi and Rakesh Kumar Mahajan. 2012. "Interactions of Phenothiazine Drugs with Bile Salts: Micellization and Binding Studies." Journal of Colloid and Interface Science 387(1):194–204.

Matsuoka, Keisuke and Yoshikiyo Moroi. 2002. "Micelle Formation of Sodium Deoxycholate and Sodium Ursodeoxycholate (Part 1)." Biochimica et Biophysica Acta - Molecular and Cell Biology of Lipids 1580(2–3):189–99.

Miyajima, K., Yokoi, M., Komatsu, H., & Nakagaki, M. 1986. "Interaction of β-Cyclodextrin with Bile Salts in Aqueous Solutions." Chemical and Pharmaceutical Bulletin 34(3):1395-1398.

Mukherjee, Bedachhanda, Aijaz Ahmad Dar, Parvaiz Ahmad Bhat, Satya Priya Moulik, and Akhil Ranjan Das. 2016. "Micellization and Adsorption Behaviour of Bile Salt Systems." RSC Advances 6(3):1769–81.

Nakashima, Toshio, Tomoyuki Anno, Hiroshi Kanda, Yuka Sato, Tatsuaki Kuroi, Hironari Fujii, Shigemi Nagadome, and Gohsuke Sugihara. 2002. "Potentiometric Study on Critical Micellization Concentrations (CMC) of Sodium Salts of Bile Acids and Their Amino Acid Derivatives." Colloids and Surfaces B: Biointerfaces 24(2):103–10.

Ninomiya, Ryoko, Keisuke Matsuoka, and Yoshikiyo Moroi. 2003. "Micelle Formation of Sodium Chenodeoxycholate and Solubilization into the Micelles: Comparison with Other Unconjugated Bile Salts." Biochimica et Biophysica Acta - Molecular and Cell Biology of Lipids 1634(3):116–25.

Olesen, Niels Erik, Peter Westh, and René Holm. 2015. “Determination of Thermodynamic Potentials and the Aggregation Number for Micelles with the Mass-Action Model by Isothermal Titration Calorimetry: A Case Study on Bile Salts.” Journal of Colloid and Interface Science 453:79–89.

Poša, Mihalj, Srcrossed Bjedov, Dušan Škorić, and Marija Sakač. 2015. “Micellization Parameters (Number Average, Aggregation Number and Critical Micellar Concentration) of Bile Salt 3 and 7 Ethylidene Derivatives: Role of the Steroidal Skeleton II.” Biochimica et Biophysica Acta - General Subjects 1850(7):1345–53.

Poša, Mihalj, Dejan Ćirin, and Veljko Krstonošić. 2013. “Physico-Chemical Properties of Bile Salt-Tween 80 Mixed Micelles in the Viewpoint of Regular Solution Theory.” Chemical Engineering Science 98:195–202.

Roda, A., A. F. Hofmann, and K. J. Mysels. 1983. “The Influence of Bile Salt Structure on Self-Association in Aqueous Solutions.” Journal of Biological Chemistry 258(10):6362–70.

Rub, Malik Abdul, Mohmad Shafi Sheikh, Abdullah M. Asiri, Naved Azum, Anish Khan, Aftab Aslam Parwaz Khan, Sher Bahadar Khan, and Kabir-Ud-Din. 2013. “Aggregation Behaviour of Amphiphilic Drug and Bile Salt Mixtures at Different Compositions and Temperatures.” Journal of Chemical Thermodynamics 64:28–39.

Rub, Malik Abdul, Mohmad Shafi Sheikh, Farah Khan, Sher Bahadar Khan, and Abdullah M. Asiri. 2014. “Bile Salts Aggregation Behavior at Various Temperatures under the Influence of Amphiphilic Drug Imipramine Hydrochloride in Aqueous Medium.” Zeitschrift Fur Physikalische Chemie 228(6–7):747–67.

Small, D. M. 1971. The Physical Chemistry of Cholanic Acids.

Subuddhi, Usharani and Ashok K. Mishra. 2007. “Micellization of Bile Salts in Aqueous Medium: A Fluorescence Study.” Colloids and Surfaces B: Biointerfaces 57(1):102–7.

Sugihara, Gohsuke and Mitsuru Tanaka. 1976. “A PH and PNa Study of Aqueous Solutions of Sodium Deoxycholate.” Bulletin of the Chemical Society of Japan 49(12):3457–60.

Yadav, Sanjay Kumar, Kushan Parikh, and Sanjeev Kumar. 2017. “Mixed Micelle Formation of Cationic Gemini Surfactant with Anionic Bile Salt: A PAH Solubilization Study.” Colloids and Surfaces A: Physicochemical and Engineering Aspects 522:105–12.

Table S2 β values of binary mixtures of bile salts.

System	Temperature [K]	β from literature data	Reference
PU:PC	283.15	0.43	(Mukherjee, Dar, Bhat, Moulik, & Das, 2016)
PU:PC	293.15	0.89	(Mukherjee et al., 2016)
PU:PC	298.15	1.32	(Azum, Rub, & Asiri, 2019)
PU:PC	298.15	2.19	(Azum et al., 2019)
PU:PC	298.15	1.39	(Azum et al., 2019)
PU:PC	298.15	1.48	(Azum et al., 2019)
PU:PC	303.15	1.14	(Mukherjee et al., 2016)
PU:PC	313.15	0.28	(Mukherjee et al., 2016)
PU:SC	283.15	-0.84	(Mukherjee et al., 2016)
PU:SC	293.15	-0.56	(Mukherjee et al., 2016)
PU:SC	303.15	-2.51	(Mukherjee et al., 2016)
PU:SC	313.15	-1.76	(Mukherjee et al., 2016)
PU:SU	283.15	-1.71	(Mukherjee et al., 2016)
PU:SU	293.15	-1.36	(Mukherjee et al., 2016)
PU:SU	298.15	-0.41	(Azum et al., 2019)
PU:SU	298.15	-0.31	(Azum et al., 2019)
PU:SU	298.15	-0.41	(Azum et al., 2019)
PU:SU	298.15	-0.85	(Azum et al., 2019)
PU:SU	303.15	-0.03	(Mukherjee et al., 2016)
PU:SU	313.15	0.84	(Mukherjee et al., 2016)
SU:PC	283.15	0.31	(Mukherjee et al., 2016)
SU:PC	293.15	-0.90	(Mukherjee et al., 2016)
SU:PC	303.15	0.77	(Mukherjee et al., 2016)
SU:PC	310.15	-0.54	(Najar, Chat, Dar, & Rather, 2013)
SU:PC	313.15	0.64	(Mukherjee et al., 2016)
PC:SC	283.15	-0.60	(Mukherjee et al., 2016)
PC:SC	293.15	-3.08	(Mukherjee et al., 2016)
PC:SC	303.15	-3.46	(Mukherjee et al., 2016)
PC:SC	313.15	-2.36	(Mukherjee et al., 2016)
SC:SU	283.15	0.28	(Mukherjee et al., 2016)
SC:SU	293.15	0.71	(Mukherjee et al., 2016)
SC:SU	303.15	-0.16	(Mukherjee et al., 2016)
SC:SU	313.15	1.10	(Mukherjee et al., 2016)

References for Table S2

Azum, N., Rub, M. A., & Asiri, A. M. (2019). Bile salt–bile salt interaction in mixed monolayer and mixed micelle formation. *Journal of Chemical Thermodynamics*, *128*, 406–414. https://doi.org/10.1016/j.jct.2018.08.030

Mukherjee, B., Dar, A. A., Bhat, P. A., Moulik, S. P., & Das, A. R. (2016). Micellization and adsorption behaviour of bile salt systems. *RSC Advances*, *6*(3), 1769–1781. https://doi.org/10.1039/c5ra20909a

Najar, M. H., Chat, O. A., Dar, A. A., & Rather, G. M. (2013). Mixed micellization and mixed

monolayer formation of sodium cholate and sodium deoxycholate in presence of hydrophobic salts under physiological conditions. *Journal of Surfactants and Detergents*, *16*(6), 967–973. https://doi.org/10.1007/s11743-013-1443-7

Table S3 Aggregation numbers of BS

Bile salt	Temp [K]	Concentration [mM]	CMC	NaCl	pH	Aggregation number	Reference
NaGC	283.15	12.80	13.20	0.00	7.00	4.10	(Olesen, Westh, & Holm, 2015)
NaGC	291.15	11.70	7.54	0.00	7.00	4.60	(Olesen et al., 2015)
NaGC	298.15	6.30	7.72	0.00	7.00	4.10	(Olesen et al., 2015)
NaGC	298.15	20.00	7.72	150.00	7.50	8.70	(Matsuoka, Maeda, & Moroi, 2003)
NaGC	310.15	10.00	14.40	120.00	7.00	5.60	(Mustan, Ivanova, Madjarova, Tcholakova, & Denkov, 2015)
NaGC	310.15	14.10	14.40	0.00	7.00	6.70	(Olesen et al., 2015)
NaGC	323.15	15.00	15.50	0.00	7.00	6.10	(Olesen et al., 2015)
NaTC	273.15	6.30	6.40	0.00	7.00	2.42	(Coello, Meijide, Rodríguez Núñez, & Vázquez Tato, 1996)
NaTC	273.15	6.30	6.40	0.00	7.00	2.44	(Coello et al., 1996)
NaTC	273.15	6.30	6.40	0.00	7.00	2.46	(Coello et al., 1996)
NaTC	273.15	6.30	6.40	0.00	7.00	2.52	(Coello et al., 1996)
NaTC	273.15	6.30	6.40	0.00	7.00	2.54	(Coello et al., 1996)
NaTC	273.15	18.00	6.40	0.00	7.00	2.59	(Coello et al., 1996)
NaTC	273.15	6.30	6.40	0.00	7.00	2.65	(Coello et al., 1996)
NaTC	273.15	6.30	6.40	0.00	7.00	2.68	(Coello et al., 1996)
NaTC	273.15	6.30	6.40	0.00	7.00	2.69	(Coello et al., 1996)
NaTC	273.15	6.30	6.40	0.00	7.00	2.69	(Coello et al., 1996)
NaTC	273.15	6.30	6.40	0.00	7.00	2.77	(Coello et al., 1996)
NaTC	283.15	8.30	6.50	0.00	7.00	2.70	(Olesen et al., 2015)
NaTC	291.15	7.40	5.02	0.00	7.00	3.80	(Olesen et al., 2015)
NaTC	298.15	5.00	5.00	0.00	7.00	3.00	(Olesen et al., 2015)
NaTC	298.15	20.00	5.00	150.00	7.50	6.00	(Matsuoka et al., 2003)
NaTC	298.20	5.00	5.00	0.00	7.00	5.00	(Matsuoka & Yamamoto, 2017)
NaTC	310.00	10.00	13.50	120.00	7.00	4.50	(Mustan et al., 2015)
NaTC	310.15	12.60	13.50	0.00	7.00	5.00	(Olesen et al., 2015)
NaTC	323.15	14.10	10.80	0.00	7.00	7.30	(Olesen et al., 2015)
NaC	273.15	19.70	5.02	0.00	7.00	3.09	(Coello et al., 1996)
NaC	283.15	17.90	12.50	0.00	7.00	5.50	(Olesen et al., 2015)
NaC	283.80	12.60	12.50	0.00	7.50	4.70	(Garidel, Hildebrand, Neubert, & Blume, 2000)
NaC	284.30	12.50	12.50	100.00	7.50	5.50	(Garidel et al., 2000)
NaC	291.15	14.40	9.24	0.00	7.00	6.20	(Olesen et al., 2015)
NaC	298.15	4.00	4.07	0.00	7.00	3.00	(Maldonado-Valderrama, Wilde, MacIerzanka, & MacKie, 2011)
NaC	298.15	9.70	4.07	0.00	7.00	4.80	(Olesen et al., 2015)
NaC	298.15	20.00	4.07	0.00	7.00	7.00	(Matsuoka & Moroi, 2002)
NaC	298.15	20.00	4.07	0.00	7.00	8.00	(Maldonado-Valderrama et al., 2011)
NaC	298.15	9.00	4.07	0.00	7.00	9.00	(Abdul Rub, Azum, & Asiri, 2017)
NaC	310.00	10.00	8.20	120.00	7.00	4.80	(Mustan et al., 2015)
NaC	310.15	18.80	8.20	0.00	7.00	6.20	(Olesen et al., 2015)

NaC	323.15	17.50	10.15	0.00	7.00	5.20	(Olesen et al., 2015)
NaC	327.90	14.00	10.30	100.00	7.50	5.40	(Garidel et al., 2000)
NaC	328.10	18.30	10.30	0.00	7.50	6.10	(Garidel et al., 2000)
NaGDC	291.15	4.40	2.80	0.00	7.00	6.00	(Olesen et al., 2015)
NaGDC	298.15	3.30	2.86	0.00	7.00	7.60	(Olesen et al., 2015)
NaGDC	310.15	5.80	5.90	0.00	7.00	7.40	(Olesen et al., 2015)
NaGDC	323.15	6.10	6.35	0.00	7.00	6.60	(Olesen et al., 2015)
NaTDC	273.15	3.00	2.95	0.00	7.00	2.92	(Coello et al., 1996)
NaTDC	273.15	3.00	2.95	0.00	7.00	4.87	(Coello et al., 1996)
NaTDC	273.15	3.00	2.95	0.00	7.00	4.96	(Coello et al., 1996)
NaTDC	273.15	3.00	2.95	0.00	7.00	5.15	(Coello et al., 1996)
NaTDC	273.15	3.00	2.95	0.00	7.00	5.28	(Coello et al., 1996)
NaTDC	273.15	7.00	2.95	0.00	7.00	5.42	(Coello et al., 1996)
NaTDC	273.15	3.00	2.95	0.00	7.00	5.44	(Coello et al., 1996)
NaTDC	273.15	3.00	2.95	0.00	7.00	5.61	(Coello et al., 1996)
NaTDC	273.15	3.00	2.95	0.00	7.00	5.79	(Coello et al., 1996)
NaTDC	273.15	3.00	2.95	0.00	7.00	6.01	(Coello et al., 1996)
NaTDC	273.15	3.00	2.95	0.00	7.00	6.22	(Coello et al., 1996)
NaTDC	283.15	4.50	3.02	0.00	7.00	7.00	(Olesen et al., 2015)
NaTDC	291.15	3.00	2.97	0.00	7.00	5.20	(Olesen et al., 2015)
NaTDC	298.15	2.30	3.72	0.00	7.00	6.70	(Olesen et al., 2015)
NaTDC	298.20	3.72	372.00	0.00	7.00	9.00	(Matsuoka & Yamamoto, 2017)
NaTDC	310.15	4.50	13.15	0.00	7.00	8.00	(Olesen et al., 2015)
NaTDC	323.15	5.20	4.20	0.00	7.00	7.10	(Olesen et al., 2015)
NaDC	273.15	4.24	4.30	0.00	7.00	5.78	(Coello et al., 1996)
NaDC	273.15	4.24	4.30	0.00	7.00	5.79	(Coello et al., 1996)
NaDC	273.15	4.24	4.30	0.00	7.00	5.80	(Coello et al., 1996)
NaDC	273.15	4.24	4.30	0.00	7.00	5.80	(Coello et al., 1996)
NaDC	273.15	13.00	4.30	0.00	7.00	5.82	(Coello et al., 1996)
NaDC	273.15	4.24	4.30	0.00	7.00	5.84	(Coello et al., 1996)
NaDC	273.15	4.24	4.30	0.00	7.00	5.85	(Coello et al., 1996)
NaDC	273.15	4.24	4.30	0.00	7.00	5.85	(Coello et al., 1996)
NaDC	273.15	4.24	4.30	0.00	7.00	5.86	(Coello et al., 1996)
NaDC	273.15	4.24	4.30	0.00	7.00	5.86	(Coello et al., 1996)
NaDC	273.15	4.24	4.30	0.00	7.00	6.74	(Coello et al., 1996)
NaDC	283.15	5.10	4.40	0.00	7.00	5.10	(Olesen et al., 2015)
NaDC	285.80	4.40	4.43	0.00	7.50	7.00	(Garidel et al., 2000)
NaDC	291.15	7.10	3.67	0.00	7.00	7.10	(Olesen et al., 2015)
NaDC	298.15	3.10	4.07	0.00	7.00	3.10	(Olesen et al., 2015)
NaDC	298.15	2.00	4.07	0.00	7.00	6.00	(Maldonado-Valderrama et al., 2011)
NaDC	298.15	20.00	4.07	0.00	7.00	10.00	(Matsuoka & Moroi, 2002)
NaDC	310.15	7.00	8.20	0.00	7.00	7.00	(Olesen et al., 2015)

NaDC	323.15	6.40	10.15	0.00	7.00	6.40	(Olesen et al., 2015)
NaDC	327.80	4.00	10.30	100.00	7.50	7.30	(Garidel et al., 2000)
NaDC	328.20	10.15	10.30	0.00	7.50	5.40	(Garidel et al., 2000)
NaGC	283.15	12.80	13.20	0.00	7.00	4.10	(Olesen et al., 2015)
NaGC	291.15	11.70	7.54	0.00	7.00	4.60	(Olesen et al., 2015)
NaGC	298.15	6.30	7.72	0.00	7.00	4.10	(Olesen et al., 2015)
NaGC	298.15	20.00	7.72	150.00	7.50	8.70	(Matsuoka et al., 2003)
NaGC	310.15	10.00	14.40	120.00	7.00	5.60	(Mustan et al., 2015)
NaGC	310.15	14.10	14.40	0.00	7.00	6.70	(Olesen et al., 2015)
NaGC	323.15	15.00	15.50	0.00	7.00	6.10	(Olesen et al., 2015)
NaTC	273.15	6.30	6.40	0.00	7.00	2.42	(Coello et al., 1996)
NaTC	273.15	6.30	6.40	0.00	7.00	2.44	(Coello et al., 1996)
NaTC	273.15	6.30	6.40	0.00	7.00	2.46	(Coello et al., 1996)
NaTC	273.15	6.30	6.40	0.00	7.00	2.52	(Coello et al., 1996)
NaTC	273.15	6.30	6.40	0.00	7.00	2.54	(Coello et al., 1996)
NaTC	273.15	18.00	6.40	0.00	7.00	2.59	(Coello et al., 1996)
NaTC	273.15	6.30	6.40	0.00	7.00	2.65	(Coello et al., 1996)
NaTC	273.15	6.30	6.40	0.00	7.00	2.68	(Coello et al., 1996)
NaTC	273.15	6.30	6.40	0.00	7.00	2.69	(Coello et al., 1996)
NaTC	273.15	6.30	6.40	0.00	7.00	2.69	(Coello et al., 1996)
NaTC	273.15	6.30	6.40	0.00	7.00	2.77	(Coello et al., 1996)
NaTC	283.15	8.30	6.50	0.00	7.00	2.70	(Olesen et al., 2015)
NaTC	291.15	7.40	5.02	0.00	7.00	3.80	(Olesen et al., 2015)
NaTC	298.15	5.00	5.00	0.00	7.00	3.00	(Olesen et al., 2015)
NaTC	298.15	20.00	5.00	150.00	7.50	6.00	(Matsuoka et al., 2003)
NaTC	298.20	5.00	5.00	0.00	7.00	5.00	(Matsuoka & Yamamoto, 2017)
NaTC	310.00	10.00	13.50	120.00	7.00	4.50	(Mustan et al., 2015)
NaTC	310.15	12.60	13.50	0.00	7.00	5.00	(Olesen et al., 2015)
NaTC	323.15	14.10	10.80	0.00	7.00	7.30	(Olesen et al., 2015)
NaC	273.15	19.70	5.02	0.00	7.00	3.09	(Coello et al., 1996)
NaC	283.15	17.90	12.50	0.00	7.00	5.50	(Olesen et al., 2015)
NaC	283.80	12.60	12.50	0.00	7.50	4.70	(Garidel et al., 2000)
NaC	284.30	12.50	12.50	100.00	7.50	5.50	(Garidel et al., 2000)
NaC	291.15	14.40	9.24	0.00	7.00	6.20	(Olesen et al., 2015)
NaC	298.15	4.00	4.07	0.00	7.00	3.00	(Maldonado-Valderrama et al., 2011)
NaC	298.15	9.70	4.07	0.00	7.00	4.80	(Olesen et al., 2015)
NaC	298.15	20.00	4.07	0.00	7.00	7.00	(Matsuoka & Moroi, 2002)
NaC	298.15	20.00	4.07	0.00	7.00	8.00	(Maldonado-Valderrama et al., 2011)
NaC	298.15	9.00	4.07	0.00	7.00	9.00	(Abdul Rub et al., 2017)
NaC	310.00	10.00	8.20	120.00	7.00	4.80	(Mustan et al., 2015)
NaC	310.15	18.80	8.20	0.00	7.00	6.20	(Olesen et al., 2015)
NaC	323.15	17.50	10.15	0.00	7.00	5.20	(Olesen et al., 2015)

NaC	327.90	14.00	10.30	100.00	7.50	5.40	(Garidel et al., 2000)
NaC	328.10	18.30	10.30	0.00	7.50	6.10	(Garidel et al., 2000)
NaGDC	291.15	4.40	2.80	0.00	7.00	6.00	(Olesen et al., 2015)
NaGDC	298.15	3.30	2.86	0.00	7.00	7.60	(Olesen et al., 2015)
NaGDC	310.15	5.80	5.90	0.00	7.00	7.40	(Olesen et al., 2015)
NaGDC	323.15	6.10	6.35	0.00	7.00	6.60	(Olesen et al., 2015)
NaTDC	273.15	3.00	2.95	0.00	7.00	2.92	(Coello et al., 1996)
NaTDC	273.15	3.00	2.95	0.00	7.00	4.87	(Coello et al., 1996)
NaTDC	273.15	3.00	2.95	0.00	7.00	4.96	(Coello et al., 1996)
NaTDC	273.15	3.00	2.95	0.00	7.00	5.15	(Coello et al., 1996)
NaTDC	273.15	3.00	2.95	0.00	7.00	5.28	(Coello et al., 1996)
NaTDC	273.15	7.00	2.95	0.00	7.00	5.42	(Coello et al., 1996)
NaTDC	273.15	3.00	2.95	0.00	7.00	5.44	(Coello et al., 1996)
NaTDC	273.15	3.00	2.95	0.00	7.00	5.61	(Coello et al., 1996)
NaTDC	273.15	3.00	2.95	0.00	7.00	5.79	(Coello et al., 1996)
NaTDC	273.15	3.00	2.95	0.00	7.00	6.01	(Coello et al., 1996)
NaTDC	273.15	3.00	2.95	0.00	7.00	6.22	(Coello et al., 1996)
NaTDC	283.15	4.50	3.02	0.00	7.00	7.00	(Olesen et al., 2015)
NaTDC	291.15	3.00	2.97	0.00	7.00	5.20	(Olesen et al., 2015)
NaTDC	298.15	2.30	3.72	0.00	7.00	6.70	(Olesen et al., 2015)
NaTDC	298.20	3.72	3.72	0.00	7.00	9.00	(Matsuoka & Yamamoto, 2017)
NaTDC	310.15	4.50	13.15	0.00	7.00	8.00	(Olesen et al., 2015)
NaTDC	323.15	5.20	4.20	0.00	7.00	7.10	(Olesen et al., 2015)
NaDC	273.15	4.24	4.30	0.00	7.00	5.78	(Coello et al., 1996)
NaDC	273.15	4.24	4.30	0.00	7.00	5.79	(Coello et al., 1996)
NaDC	273.15	4.24	4.30	0.00	7.00	5.80	(Coello et al., 1996)
NaDC	273.15	4.24	4.30	0.00	7.00	5.80	(Coello et al., 1996)
NaDC	273.15	13.00	4.30	0.00	7.00	5.82	(Coello et al., 1996)
NaDC	273.15	4.24	4.30	0.00	7.00	5.84	(Coello et al., 1996)
NaDC	273.15	4.24	4.30	0.00	7.00	5.85	(Coello et al., 1996)
NaDC	273.15	4.24	4.30	0.00	7.00	5.85	(Coello et al., 1996)
NaDC	273.15	4.24	4.30	0.00	7.00	5.86	(Coello et al., 1996)
NaDC	273.15	4.24	4.30	0.00	7.00	5.86	(Coello et al., 1996)
NaDC	273.15	4.24	4.30	0.00	7.00	6.74	(Coello et al., 1996)
NaDC	283.15	5.10	4.40	0.00	7.00	5.10	(Olesen et al., 2015)
NaDC	285.80	4.40	4.43	0.00	7.50	7.00	(Garidel et al., 2000)
NaDC	291.15	7.10	3.67	0.00	7.00	7.10	(Olesen et al., 2015)
NaDC	298.15	3.10	4.07	0.00	7.00	3.10	(Olesen et al., 2015)
NaDC	298.15	2.00	4.07	0.00	7.00	6.00	(Maldonado-Valderrama et al., 2011)
NaDC	298.15	20.00	4.07	0.00	7.00	10.00	(Matsuoka & Moroi, 2002)
NaDC	310.15	7.00	8.20	0.00	7.00	7.00	(Olesen et al., 2015)
NaDC	323.15	6.40	10.15	0.00	7.00	6.40	(Olesen et al., 2015)

NaDC	327.80	4.00	10.30	100.00	7.50	7.30	(Garidel et al., 2000)
NaDC	328.20	10.15	10.30	0.00	7.50	5.40	(Garidel et al., 2000)

References for Table S3

Abdul Rub, M., Azum, N., & Asiri, A. M. (2017). Binary Mixtures of Sodium Salt of Ibuprofen and Selected Bile Salts: Interface, Micellar, Thermodynamic, and Spectroscopic Study. *Journal of Chemical and Engineering Data*, *62*(10), 3216–3228. https://doi.org/10.1021/acs.jced.7b00298

Coello, A., Meijide, F., Rodríguez Núñez, E., & Vázquez Tato, J. V. (1996). Aggregation behavior of bile salts in aqueous solution. *Journal of Pharmaceutical Sciences*, *85*(1), 9–15. https://doi.org/10.1021/js950326j

Garidel, P., Hildebrand, A., Neubert, R., & Blume, A. (2000). Thermodynamic characterization of bile salt aggregation as a function of temperature and ionic strength using isothermal titration calorimetry. *Langmuir*, *16*(12), 5267–5275. https://doi.org/10.1021/la9912390

Maldonado-Valderrama, J., Wilde, P., MacIerzanka, A., & MacKie, A. (2011). The role of bile salts in digestion. *Advances in Colloid and Interface Science*, *165*(1), 36–46. https://doi.org/10.1016/j.cis.2010.12.002

Matsuoka, K., Maeda, M., & Moroi, Y. (2003). Micelle formation of sodium glyco- and taurocholates and sodium glyco- and taurodeoxycholates and solubilization of cholesterol into their micelles. *Colloids and Surfaces B: Biointerfaces*, *32*(2), 87–95. https://doi.org/10.1016/S0927-7765(03)00148-6

Matsuoka, K., & Moroi, Y. (2002). Micelle formation of sodium deoxycholate and sodium ursodeoxycholate (Part 1). *Biochimica et Biophysica Acta - Molecular and Cell Biology of Lipids*, *1580*(2–3), 189–199. https://doi.org/10.1016/S1388-1981(01)00203-7

Matsuoka, K., & Yamamoto, A. (2017). Study on micelle formation of bile salt using nuclear magnetic resonance spectroscopy. *Journal of Oleo Science*, *66*(10), 1129–1137. https://doi.org/10.5650/jos.ess17063

Mustan, F., Ivanova, A., Madjarova, G., Tcholakova, S., & Denkov, N. (2015). Molecular Dynamics Simulation of the Aggregation Patterns in Aqueous Solutions of Bile Salts at Physiological Conditions. *Journal of Physical Chemistry B*, *119*(51), 15631–15643. https://doi.org/10.1021/acs.jpcb.5b07063

Olesen, N. E., Westh, P., & Holm, R. (2015). Determination of thermodynamic potentials and the aggregation number for micelles with the mass-action model by isothermal titration calorimetry: A case study on bile salts. *Journal of Colloid and Interface Science*, *453*, 79–89. https://doi.org/10.1016/j.jcis.2015.03.069

Table S4 MSR values for BSs with given solubilizates

BS	Solubilizate	logKow	Volume	Temp [K]	pH	Na^+ [M]	MSR	Reference
TDC	Azobenzene	4.13	174.56	310.15	6.30	15.00	0.04	(Hofmann, 1963)
TCDC	Azobenzene	4.13	174.56	310.15	6.30	15.00	0.03	(Hofmann, 1963)
TC	Azobenzene	4.13	174.56	310.15	6.30	15.00	0.02	(Hofmann, 1963)
GDC	Azobenzene	4.13	174.56	310.15	6.30	15.00	0.05	(Hofmann, 1963)
GCDC	Azobenzene	4.13	174.56	310.15	6.30	15.00	0.04	(Hofmann, 1963)
GC	Azobenzene	4.13	174.56	310.15	6.30	15.00	0.02	(Hofmann, 1963)
TDC	Monoolein	6.61	386.27	310.15	6.30	0.15	1.41	(Hofmann, 1963)
TCDC	Monoolein	6.61	386.27	310.15	6.30	0.15	1.58	(Hofmann, 1963)
TC	Monoolein	6.61	386.27	310.15	6.30	0.15	0.84	(Hofmann, 1963)
GDC	Monoolein	6.61	386.27	310.15	6.30	0.15	1.76	(Hofmann, 1963)
GCDC	Monoolein	6.61	386.27	310.15	6.30	0.15	1.90	(Hofmann, 1963)
GC	Monoolein	6.61	386.27	310.15	6.30	0.15	1.42	(Hofmann, 1963)
GDC	vit. K	8.80	483.87	298.15	7.00	0.00	0.03	(Nagata, Yotsuyanagi, & Ikeda, 1988)
GC	vit. K	8.80	483.87	298.15	7.00	0.00	0.02	(Nagata et al., 1988)
GDC	vit. K	8.80	483.87	298.15	7.50	0.00	0.03	(Nagata et al., 1988)
GC	vit. K	8.80	483.87	298.15	7.50	0.00	0.02	(Nagata et al., 1988)
GDC	cholestrol	7.62	423.13	310.15	7.00	0.00	0.46	(Neiderhiser & Roth, 1968)
TDC	cholestrol	7.62	423.13	310.15	7.00	0.00	0.37	(Neiderhiser & Roth, 1968)
GC	cholestrol	7.62	423.13	310.15	7.00	0.00	0.36	(Neiderhiser & Roth, 1968)
GCDC	cholestrol	7.62	423.13	310.15	7.00	0.00	0.29	(Neiderhiser & Roth, 1968)
TC	cholestrol	7.62	423.13	310.15	7.00	0.00	0.27	(Neiderhiser & Roth, 1968)
TCDC	cholestrol	7.62	423.13	310.15	7.00	0.00	0.23	(Neiderhiser & Roth, 1968)
C	Benzene	1.94	84.04	298.00	7.00	0.00	0.90	(Kolehmainen, 1985)
C	Fluorobenzene	2.10	88.97	298.00	7.00	0.00	0.45	(Kolehmainen, 1985)
C	Hexafluorobenzene	2.63	113.63	298.00	7.00	0.00	0.55	(Kolehmainen, 1985)
C	Toluene	2.39	100.60	298.00	7.00	0.00	0.45	(Kolehmainen, 1985)
C	p-Fluorotoulene	2.55	105.54	298.00	7.00	0.00	0.45	(Kolehmainen, 1985)
C	Styrene	2.79	111.78	298.00	7.00	0.00	0.60	(Kolehmainen, 1985)
C	propenylbenzene	3.04	128.02	298.00	7.00	0.00	0.40	(Kolehmainen, 1985)
C	Anisole	1.99	109.59	298.00	7.00	0.00	0.50	(Kolehmainen, 1985)
C	Fluoroanisole	2.11	114.52	298.00	7.00	0.00	1.00	(Kolehmainen, 1985)
C	Acetophenone	1.84	119.59	298.00	7.00	0.00	0.80	(Kolehmainen, 1985)
C	Fluoroacetophenone	1.98	124.52	298.00	7.00	0.00	0.50	(Kolehmainen, 1985)
C	Nitrobenzene	1.90	107.38	298.00	7.00	0.00	0.25	(Kolehmainen, 1985)
C	Mesitylene	3.21	133.73	298.00	7.00	0.00	0.35	(Kolehmainen, 1985)
C	Tetraline	3.15	140.41	298.00	7.00	0.00	1.30	(Kolehmainen, 1985)
C	Veatrole	1.61	135.13	298.00	7.00	0.00	1.45	(Kolehmainen, 1985)
DC	Benzene	1.94	84.04	298.00	7.00	0.00	0.81	(Kolehmainen, 1985)
DC	Fluorobenzene	2.10	88.97	298.00	7.00	0.00	0.76	(Kolehmainen, 1985)
DC	Fluorotoluene	2.50	105.54	298.00	7.00	0.00	0.72	(Kolehmainen, 1985)
DC	Fluoroanisole	2.11	114.52	298.00	7.00	0.00	0.96	(Kolehmainen, 1985)

DC	vit. K	8.80	483.87	298.15	7.00	0.00	0.05	(Nagata et al., 1988)
C	vit. K	8.80	483.87	298.15	7.00	0.00	0.03	(Nagata et al., 1988)
DC	vit. K	8.80	483.87	298.15	7.50	0.00	0.05	(Nagata et al., 1988)
C	vit. K	8.80	483.87	298.15	7.50	0.00	0.02	(Nagata et al., 1988)
C	Cholesterol	7.62	423.13	310.15	10.00	0.00	0.04	(Nagadome, Okazaki, Lee, Sasaki, & Sugihara, 2001)
C	Stigmasterol	7.87	450.33	310.15	10.00	0.00	0.02	(Nagadome et al., 2001)
C	Cholesterol+Stigmasterol	7.70	426.73	310.15	10.00	0.00	0.04	(Nagadome et al., 2001)
C	cholestanol	7.80	429.34	310.15	10.00	0.00	0.03	(Nagadome et al., 2001)
DC	Cholesterol	7.62	423.13	310.15	10.00	0.00	0.07	(Nagadome et al., 2001)
DC	Stigmasterol	7.87	450.33	310.15	10.00	0.00	0.04	(Nagadome et al., 2001)
DC	Cholesterol+Stigmasterol	7.70	436.73	310.15	10.00	0.00	0.08	(Nagadome et al., 2001)
DC	Cholestanol	7.80	429.34	310.15	10.00	0.00	0.06	(Nagadome et al., 2001)

References for Table S4

Hofmann, A. F. (1963). The function of bile salts in fat absorption. The solvent properties of dilute micellar solutions of conjugated bile salts. *Biochemical Journal*, *89*(1), 57–68. https://doi.org/10.1042/bj0890057

Kolehmainen, E. (1985). Solubilization of aromatics in aqueous bile salts. I. benzene and alkylbenzenes in sodium cholate: 1H NMR study. *Journal of Colloid And Interface Science*, *105*(1), 273–277. https://doi.org/10.1016/0021-9797(85)90369-8

Nagadome, S., Okazaki, Y., Lee, S., Sasaki, Y., & Sugihara, G. (2001). Selective solubilization of sterols by bile salt micelles in water: A thermodynamic study. *Langmuir*, *17*(14), 4405–4412. https://doi.org/10.1021/la010087h

Nagata, M., Yotsuyanagi, T., & Ikeda, K. (1988). Solubilization of Vitamin K1 by Bile Salts and Phosphatidylcholine-Bile Salts Mixed Micelles. *Journal of Pharmacy and Pharmacology*, *40*(2), 85–88. https://doi.org/10.1111/j.2042-7158.1988.tb05186.x

Neiderhiser, D. H., & Roth, H. P. (1968). Cholesterol Solubilization by Solutions of Bile Salts and Bile Salts Plus Lecithin. *Proceedings of the Society for Experimental Biology and Medicine*, *128*(1), 221–225. https://doi.org/10.3181/00379727-128-32983

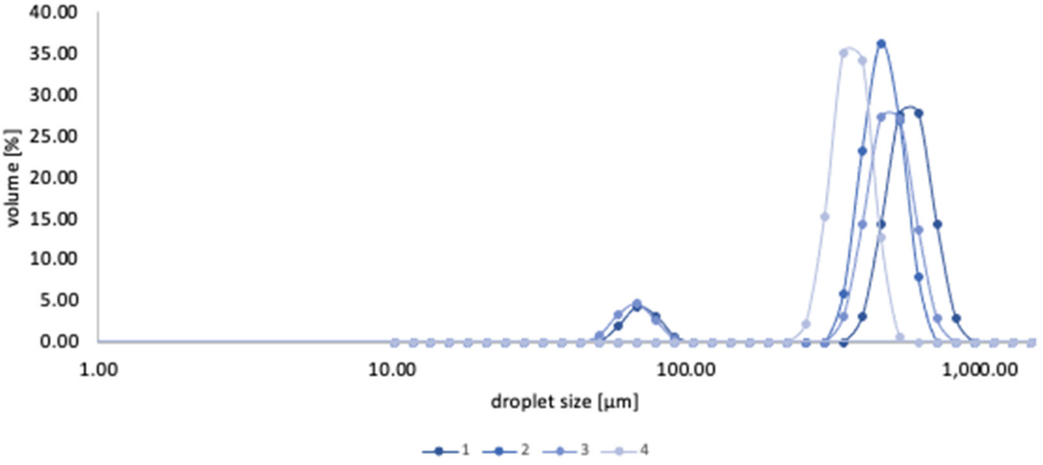

Figure S1. Particle size distribution of O/W emulsion with 0.5% WPI. Numbers: 1,2,3,4 corresponds to each number of run.

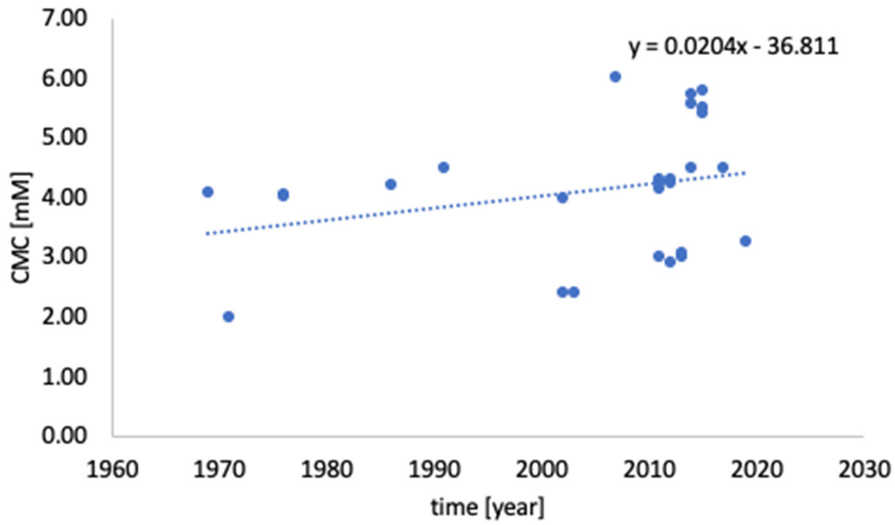

Figure S2. CMC of NaDC at 298.15K increased from 3.92 mM in the 1960-70s to an average of ~4.16 mM in the 2010-2020.
